# Pan-cancer analysis of the prognostic and immunological role of Fanconi anemia complementation group E

**DOI:** 10.3389/fgene.2022.1024989

**Published:** 2023-01-04

**Authors:** Zhixian Zhou, Huan Yin, Suye Suye, Jiarong He, Chun Fu

**Affiliations:** ^1^ Department of Obstetrics and Gynecology, Second Xiangya Hospital, Central South University, Changsha, China; ^2^ Department of Neurosurgery, Second Xiangya Hospital, Central South University, Changsha, China

**Keywords:** Fanconi anemia, *FANCE*, pan-cancer, prognosis, tumor immunity

## Abstract

Fanconi anemia (FA) genes contribute to tumorigenesis by regulating DNA repair. Despite its importance for assembly and functionality of the FA core complex, no pan-cancer analysis of *FANCE* was performed. We aimed to provide a comprehensive understanding of the role of *FANCE* in cancers. Based on The Cancer Genome Atlas (TCGA), Cancer Cell Line Encyclopedia (CCLE), Genotype Tissue-Expression (GTEx), Human Protein Atlas (HPA), Gene Expression Omnibus (GEO), and Cancer Single-cell Atlas (CancerSEA) databases, we investigated the carcinogenicity of *FANCE* using various bioinformatics methods, including *FANCE* expression and prognosis, immune invasion, tumor mutation burden, microsatellite instability, and neoantigens. We monitored *Fance* mutations in mice that caused tumorigenesis. *FANCE* expression and activity scores were upregulated in 15 and 21 cancers. High expression of *FANCE* affected shorter overall survival (OS) in seven cancers and longer overall survival in three cancers. It was correlated with shorter overall survival and progression-free interval (PFI) in endometrial cancer and longer overall survival and PFI in cervical cancer. *FANCE* expression negatively correlated with stromal/immune scores in 21 cancers including cervical cancer, endometrial cancer, and ovarian cancer. *FANCE* expression negatively correlated with CD8 T cells in endometrial cancer and positively correlated with M1 macrophages in cervical cancer, possibly related to cancer prognosis. *FANCE* positively correlated with immune checkpoint inhibitors PD-1, PD-L1, and CTLA4 in endometrial cancer and ovarian cancer. *FANCE* expression positively correlated with microsatellite instability, tumor mutational burden, and neoantigens in 7, 22, and five cancers, especially in endometrial cancer, potentially increasing the effectiveness of immunotherapy. Single-cell sequencing data showed *FANCE* was primarily expressed in cancer cells in cervical and ovarian cancer, and in fibroblasts in endometrial cancer. *Fance* heterozygous mutant mice had increased tumor incidences and shorter overall survival and tumor-free survival (TFS) than *Fance* homozygous mutant mice and wild-type mice. Conclusively, *FANCE* potential to serve as a biomarker for cancer prognosis and may predict cancer immunotherapy responses. *Fance* heterozygous mutant resulted in increased tumorigenesis and poor prognosis in mice.

## 1 Introduction

Fanconi anemia (FA) is characterized by bone marrow failure, developmental abnormalities, and cancer predisposition (Che et al., 2018). So far, 22 FA complementation groups have been identified (*FANCA/B/C/D/D2/E/F/G/I/J/L/M/N/O/P/Q/R/S/T/U/V/W*). The defection of FA genes is associated with high incidences of cancers in patients, particularly those originating from hematopoietic and squamous cells, most commonly seen in acute myeloid leukemia, pancreatic cancer, solid tumors, cervical cancer, oral cancer, prostate cancer, bone marrow failure, breast cancer, and ovarian cancer (Nepal et al., 2017). High expression of FA genes (*FANCF D1/D2/I/J/N/S*) has been demonstrated linked to poor prognosis in various cancers, including head and neck cancer (Verhagen et al., 2018), myeloid leukemia (Padella et al., 2021), liver cancer (Xu H. et al., 2021), lung cancer (Miao et al., 2022; Zhang et al., 2022), breast cancer (Fagerholm et al., 2013; Feng and Jin, 2019), cervical cancer (Liu et al., 2021), and ovarian cancer (Ding et al., 2016; Moes-Sosnowska et al., 2019). Fanconi Anemia Complementation Group E (FANCE) is a key component in the architecture of the FA core complex (FANCA/B/C/E/F/G/L/M/T) and the connection of the downstream FANCD2–FANCI monoubiquitylation complex, DNA damage repair proteins (FANCD1/J/N/O/P/Q/R/S/U/V/W), and their collaborators (FA associate proteins) (Gordon et al., 2005; Kottemann and Smogorzewska, 2013). Patients with *FANCE* defects are at risk of developing esophageal cancer, gastric cancer, and leukemia (Ali et al., 2009; Akbari et al., 2011; Li et al., 2013). *FANCE* high expression was reported consistent with poor prognosis in liver cancer (Takahashi et al., 2022). However, the mechanism that *FANCE* contributes to the development and progression of cancer is still unclear.

The tumor microenvironment can have a significant impact on tumor development, invasion, metastasis, and prognosis (Ren et al., 2018; Jiang et al., 2020; Malherbe, 2021; Bremnes et al., 2016; Oura et al., 2021). Studies showed the expression of FA genes (*FANCR/D2/I*) regulates immune infiltration and modulates the immunotherapy response in cancers. *FANCR* expression was positively correlated with inhibitory immune checkpoint proteins in liver cancer, promoting immune escape and inhibiting immunotherapy responses (Xu H. et al., 2021). *FANCD2* expression facilitates tumor immune escape in lung cancer by suppressing the immune microenvironment, while patients with high expression of *FANCD2* exhibited higher tumor mutation burden (TMB) indicating greater responses to immunotherapy (Miao et al., 2022; Zhang et al., 2022). *FANCI* expression suppressed the immune microenvironment and was positively related to infiltrated CD4 T cells, CD8 T cells, and neutrophils in cervical cancer (Liu et al., 2021). Moreover, patients with FA-related head and neck cancer benefit from immunotherapy by activating lymphocyte-mediated antitumor activity (Lee et al., 2021). It remains unclear what role of *FANCE* plays in tumor immunity. We investigate the prognostic and immunological effects of *FANCE* on various cancers.

We conducted a pan-cancer analysis of *FANCE* in 33 cancers. *FANCE* expression was analyzed with prognosis, function enrichment, tumor microenvironment (TME), tumor-infiltrating immune cells (TIICs), immune-regulated genes, immune checkpoint genes, microsatellite instability (MSI), TMB, and neoantigens. In addition, we used *Fance* mutant mice models to observe tumorigenesis. Our findings suggest that *FANCE* potentially serves as a biomarker for cancer prognosis. *FANCE* correlated with immunity infiltration and predicted the response to immunotherapy in cancers. Additionally, we identified the tumorigenesis of *Fance* mutation in mice. Our study provided preliminary insights into the role of *FANCE* in tumorigenesis, prognosis prediction, and immunotherapy. These data may serve as references to new targets for clinical treatment.

## 2 Material and methods

### 2.1 Expression and gene activity of *FANCE*

Genotype-Tissue Expression (GTEx) database (https://commonfund.nih.gov/GTEx) examined the expression of *FANCE* in 31 normal tissues. Cancer Cell Line Encyclopedia (CCLE) database (https://portals.broadinstitute.org/ccle/) was used to assess the expression of *FANCE* in 21 tumor cell lines. The Cancer Genome Atlas (TCGA) database (https://portal.gdc.cancer.gov) was used to determine the differential expression of *FANCE* between tumors and normal tissues. Single-sample gene set enrichment analysis (ssGSEA) was used to estimate the gene activity scores of *FANCE* in normal and tumor tissues. The human protein atlas (HPA) database (http://www.proteinatlas.org/) was used to evaluate the expression of *FANCE* protein in normal and tumor tissues using immunohistochemistry (IHC).

*FANCE* gene expression data from these downloaded datasets for subsequent analyses, contains 11,007 samples from 33 cancers, involving adrenocortical carcinoma (ACC, *n* = 79), bladder urothelial carcinoma (BLCA, *n* = 427), breast invasive carcinoma (BRCA, *n* = 1211), cervical squamous cell carcinoma (CESC, *n* = 309), cholangiocarcinoma (CHOL, *n* = 45), colon adenocarcinoma (COAD, *n* = 499), lymphoid neoplasm diffuse large B cell lymphoma (DLBC, *n* = 48), esophageal carcinoma (ESCA, *n* = 173), glioblastoma multiforme (GBM, *n* = 172), head and neck squamous cell carcinoma (NHSC, *n* = 546), kidney chromophobe (KICH, *n* = 89), kidney renal clear cell carcinoma (KIRC, *n* = 603), kidney renal papillary cell carcinoma (KIRP, *n* = 321), acute myeloid leukemia (LAML, *n* = 151), brain lower grade glioma (LGG, *n* = 525), liver hepatocellular carcinoma (LIHC, *n* = 423), lung adenocarcinoma (LUAD, *n* = 574), lung squamous cell carcinoma (LUSC, *n* = 550), mesothelioma (MESO, *n* = 86), ovarian serous cystadenocarcinoma (OV, *n* = 379), pancreatic adenocarcinoma (PAAD, *n* = 182), pheochromocytoma and paraganglioma (PCPG, *n* = 186), prostate adenocarcinoma (PRAD, *n* = 548), rectum adenocarcinoma (READ), sarcoma (SARC, *n* = 265), skin cutaneous melanoma (SKCM, *n* = 472), stomach adenocarcinoma (STAD, *n* = 407), testicular germ cell tumors (TGCT, *n* = 156), thyroid carcinoma (THCA, *n* = 568), thymoma (THYM, *n* = 121), uterine corpus endometrial carcinoma (UCEC, *n* = 10,872), uterine carcinosarcoma (UCS, *n* = 56), and uveal melanoma (UVM, *n* = 80). The expression data were shown according to a log2 (TPM+1) scale. Strawberry Perl (5.32.1.1) and R (4.2.0) were used to process and plot the data. R packages “plyr,” “reshape2,” “ggpubr,” “GSVA,” and “GSEABase” were used.

### 2.2 GSEA of *FANCE* expression

GSEA was performed to analyze the biological pathway of *FANCE* expression in cancers. According to the TCGA database, samples were divided into two subgroups based on the median expression level of *FANCE*. The top five pathways of Kyoto Encyclopedia of Genes and Genomes (KEGG) terms was presented. This analysis was conducted with R packages “org.Hs.eg.db,” “clusterProfiler,” “enrichplot,” and “ggplot2.”

### 2.3 Prognosis value of *FANCE* expression

The TCGA database was used to collect survival and clinical data on 33 cancers. *FANCE* expression and patient prognosis were investigated using four indicators: overall survival (OS), disease-specific survival (DSS), progression-free interval (PFI), and disease-free interval (DFI). Survival analysis was performed using Cox regression and Kaplan–Meier (KM). “survival,” “survminer,” “ggpubr,” “forestplot” packages were used.

### 2.4 *FANCE* expression and TME, TIICs, immune-regulated genes, and immune checkpoint genes

In 33 cancers based on the TCGA dataset, the stromal, immune, and ESTIMATE scores were calculated using the ESTIMATE algorithm. CIBERSORT was used to evaluate *FANCE* expression and the proportion of TIICs in 33 cancers based on the TCGA dataset. Co-expression of *FANCE* with 150 immune moderator genes and 60 immune checkpoint genes were analyzed based on the TCGA database. “ggplot2,” “ggpubr,” “ggExtra,” “limma,” “estimate,” “psych,” and “CIBERSORT” packages were used.

### 2.5 *FANCE* expression and MSI, TMB, and neoantigens

Data on somatic mutations were downloaded from TCGA. TMB and MSI grades were computed with Perl scripts. Neoantigen prediction aimed to assess factors affecting immunogenicity in tumors from the TCGA (Thorsson et al., 2018). The ScanNeo tool2 was used to measure the number of neoantigens in each tumor sample (Xu C. et al., 2021). The R packages “fmsb,” “ggradar,” and “ggplot2” were used.

### 2.6 Single-cell RNA sequencing (scRNA-seq) data analysis

ScRNA-seq data for endometrial cancer, ovarian cancer, and cervical cancer (GSE173682, GSM5236544, and GSM5236545) were downloaded from the Gene Expression Omnibus (GEO) database (https://www.ncbi.nlm.nih.gov/geo/). The Seurat package was used for normalization, feature selection, and clustering of the gene expression matrices. The selection method “vst” of Seurat was applied to pick the top 2,000 variable features. The UMAP and tSNE algorithms were applied for the dimensionality reduction of the data. The FindCluster function was used to cluster cells. SingleR was used to annotate cells automatically. The Cancer Single-cell Atlas (CancerSEA) database (https://biocc.hrbmu.edu.cn/CancerSEA/) was employed to explore the function of *FANCE*.

### 2.7 *Fanc*e gene mutant mice increased tumor susceptibility

This study has been approved by the Laboratory Animal Welfare and Ethical Committee of Central South University (IACUC Number: 2020sydw1041). *Fance* mutant mice with FVB/N backgrounds have been constructed by the research group (Fu et al., 2016a; Fu et al., 2016b). In Central South University’s Laboratory Animals Department, laboratory animals were housed under pathogen-free conditions (20–22 C, 12-h light/dark cycle). Histopathological examinations of various tissues and organs were performed after the mice monitored tumorigenesis. Paraffin-embedded sections stained with H&E were used to confirm and categorize tumors. Log-rank test was used to analyze survival. Section scanning was performed using 3DHISTECH (Hungary) (PANNORAMIC, DESK/MIDI/250/1000, CaseViewer2.4).

### 2.8 Statistical analysis

The Kruskal-Wallis test was applied to compare the expression of *FANCE* among normal tissues and tumor cell lines. The comparison of normal tissue and cancer tissue used two sets of t-tests. The KM curve, Cox proportional hazard regression model, and the log-rank test were applied to all survival analyses. Spearman’s test was used to analyze the correlation between expression and tumor immunity. Chi-square tests were used to compare tumor incidence, and Bonferroni tests were conducted to compare multiple groups. *p* < .05 was regarded as statistically significant.

## 3 Results

### 3.1 *FANCE* expression and gene activity scores

**FIGURE 1:**
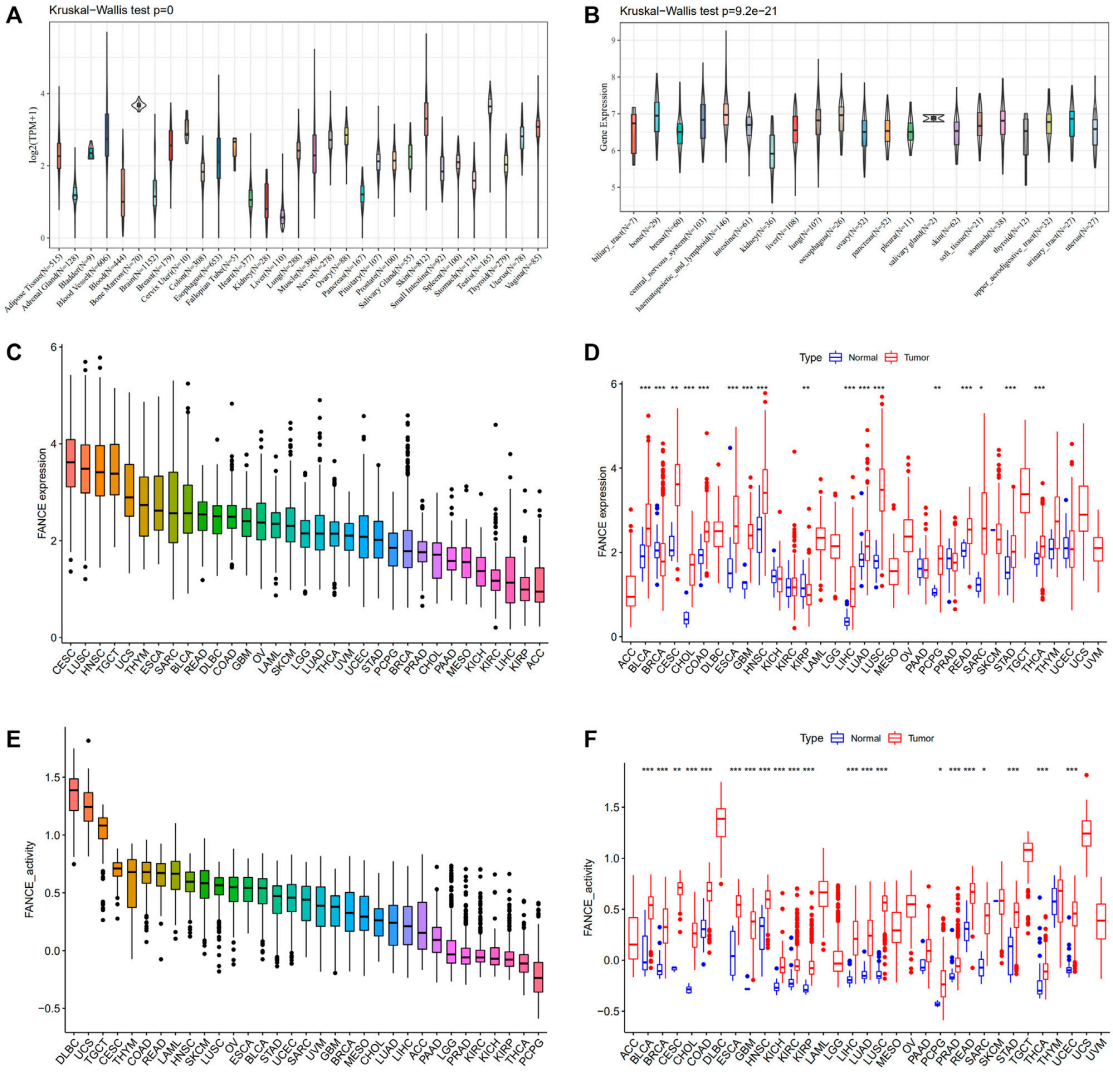
Expression and gene activity scores of *FANCE*. **(A)** *FANCE* expression in 31 normal tissues from the Genotype Tissue-Expression (GTEx) database. **(B)** *FANCE* expression in 21 tumor cell lines from the Cancer Cell Line Encyclopedia (CCLE) database. **(C)** The expression of *FANCE* in 33 cancers ranked from highest (left) to lowest (right) in the TCGA database. **(D)** The expression of *FANCE* in 33 cancers compared to normal tissues from The Cancer Genome Atlas (TCGA) database. **(E)** Gene activity scores of *FANCE* in 33 cancers ranked from highest (left) to lowest (right) based on TCGA and sngle-sample gene set enrichment analysis (ssGSEA). **(F)** Gene activity scores of *FANCE* in 33 cancers compared to normal tissues based on TCGA and ssGSEA. *p < .05; **p < .01; and ***p < .001.

We compared *FANCE* expression levels in 31 normal tissues based on the GTEx database. *FANCE* was detected highest in the testis, bone marrow, and skin (Figure 1A). All 21 tumor cell lines from the CCLE database expressed *FANCE* (Figure 1B)*.* Based on the TCGA dataset, *FANCE* expression was highest in CESC (Figure 1C). *FANCE* expression was higher in 15 tumors than in normal tissues, including CESC, ESCA, STAD, LIHC, BLCA, CHOL, COAD, GBM, HNSC, LUAD, LUSC, PCPG, READ, SARC, and THCA. BRCA and KIRP tumors had lower *FANCE* expression (Figure 1D). Gene activity scores of *FANCE* were highest in DLBC, UCS, TGCT, and were significantly higher in 21 cancers than in normal tissues, including CESC, UCEC, ESCA, STAD, LIHC, BRCA, PRAD, BLCA, CHOL, COAD, GBM, HNSC, KICH, KIRC, KIRD, LUAD, LUSC, PCPG, READ, SARC, and THCA (Figures 1E,F).

**FIGURE 2:**
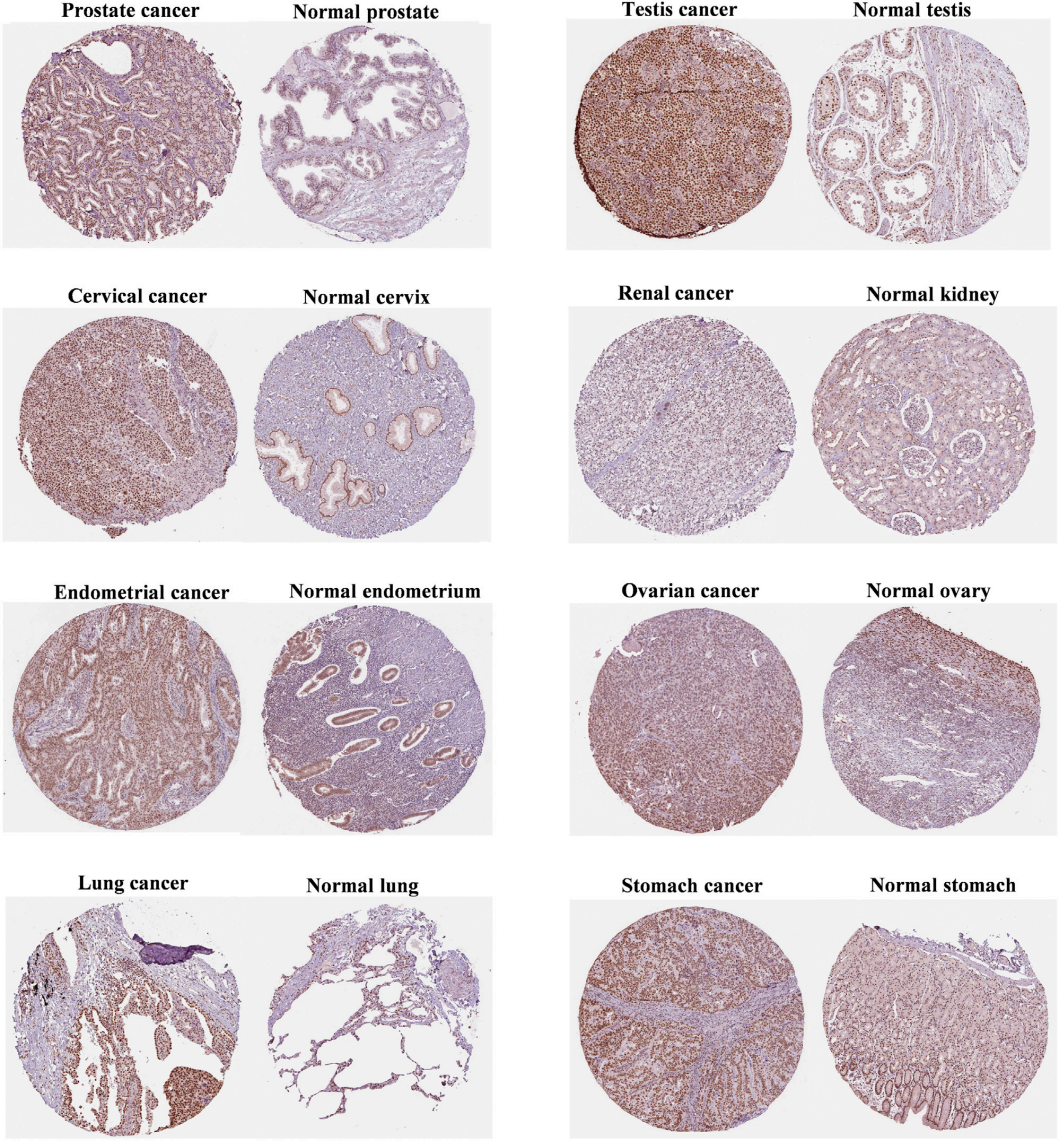
Immunohistochemistry of FANCE protein expression between tumor and normal tissues from the Human Protein Atlas (HPA) database.

As shown in Figure 2, FANCE staining was higher in prostate, testis, and cervical cancers than in normal tissues. In addition, FANCE staining was lower in renal cancer than in normal tissues. These results were consistent with gene expression. FANCE staining observed no difference in endometrial, ovarian, lung, and gastric cancers compared with normal tissue.

### 3.2 Biological functions of *FANCE* expression

**FIGURE 3:**
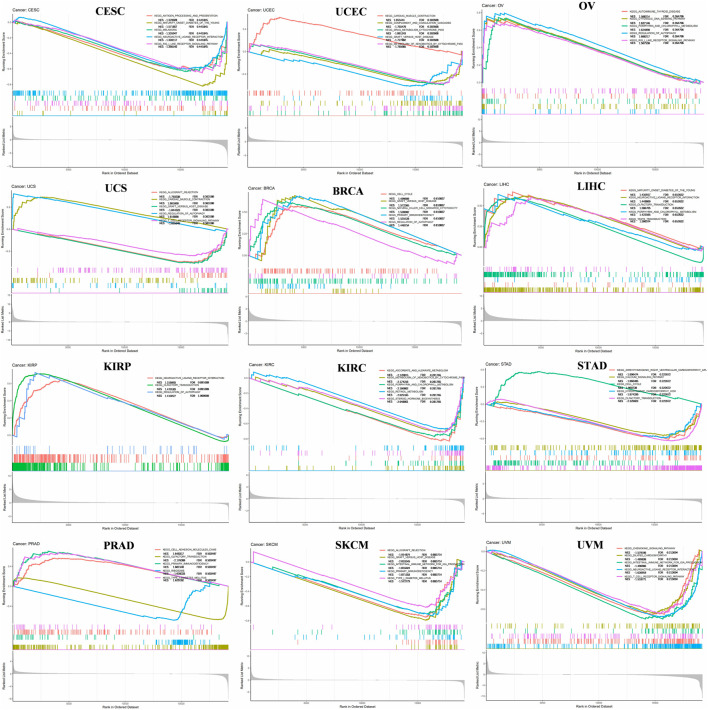
Gene set enrichment analysis (GSEA) of *FANCE* expression in cancers. The top five most relevant signaling pathways in the Kyoto Encyclopedia of Genes and Genomes (KEGG) dataset were displayed. Normalized Enrichment Score (NES) value and FDR value (qvalue) were displayed. Peaks on the upward curve indicate positive regulation and peaks on the downward curve indicate negative regulation.

*FANCE* mainly upregulated signaling pathways in 11 tumors and downregulated signaling pathways in 22 tumors (Figure 3, Supplementary Figure S1). *FANCE* negatively regulated antigen processing and presentation pathway CESC and positively regulated autoimmune thyroid disease in OV. *FANCE* inhibited DNA replication and cell cycle in UCEC. *FANCE* negatively regulated T cell receptor signaling pathway in USC. *FANCE* positively regulated cell cycle, natural killer cell mediated cytotoxicity and primary immunodeficiency in BRCA. *FANCE* positively regulated cell cycle in STAD. These results suggest that *FANCE* functions in tumors by regulating immune function and DNA replication pathways, and so on.

### 3.3 Prognostic value of *FANCE* expression

**FIGURE 4:**
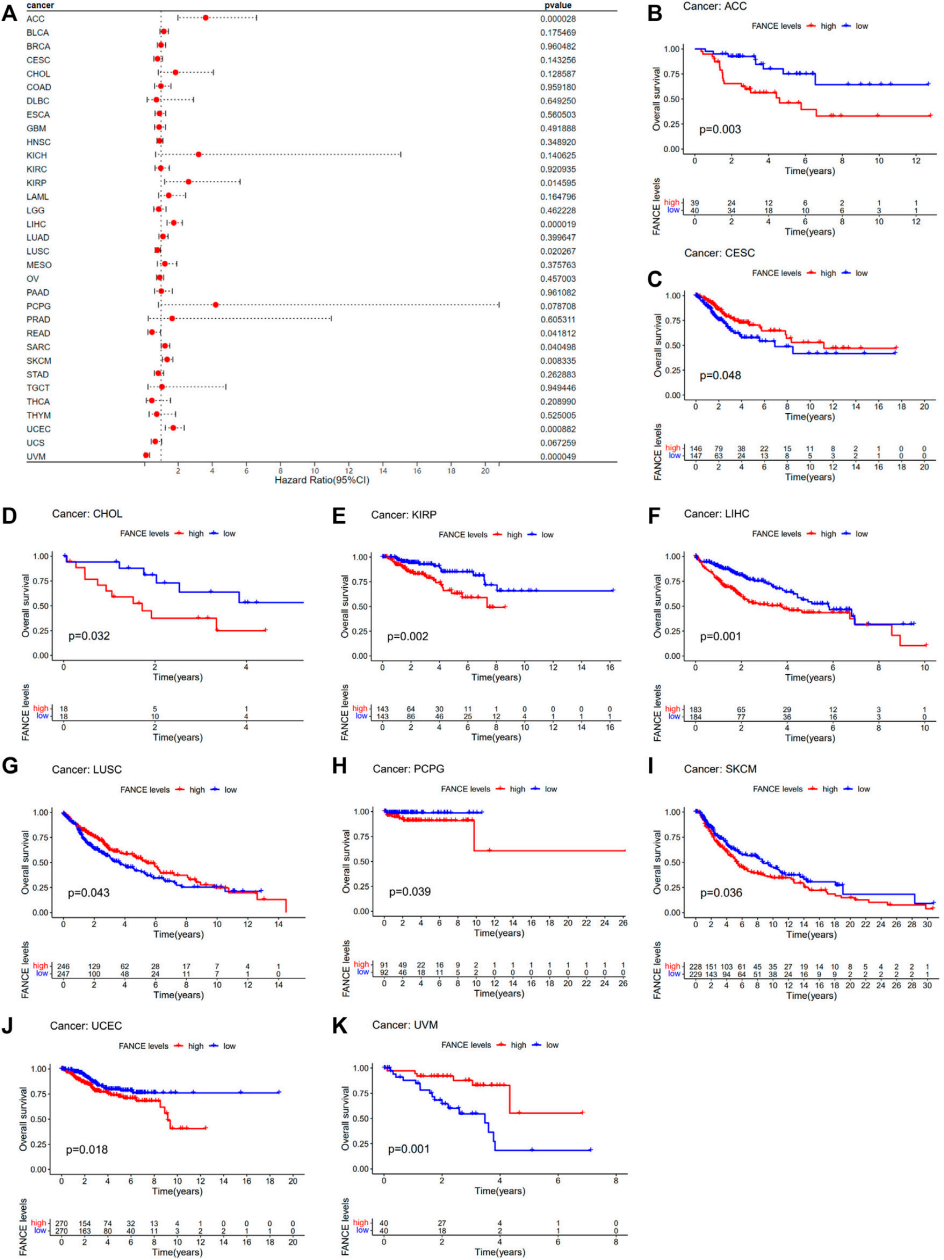
Correlation between *FANCE* expression and overall survival (OS) using data from the TGCA database. **(A)** Forest plot of *FANCE* expression and OS in 33 cancers. **(B–K)** The Kaplan–Meier survival analysis for *FANCE* expression and OS in 10 cancers. *p < .05; **p < .01; and ***p < .001.

**FIGURE 5:**
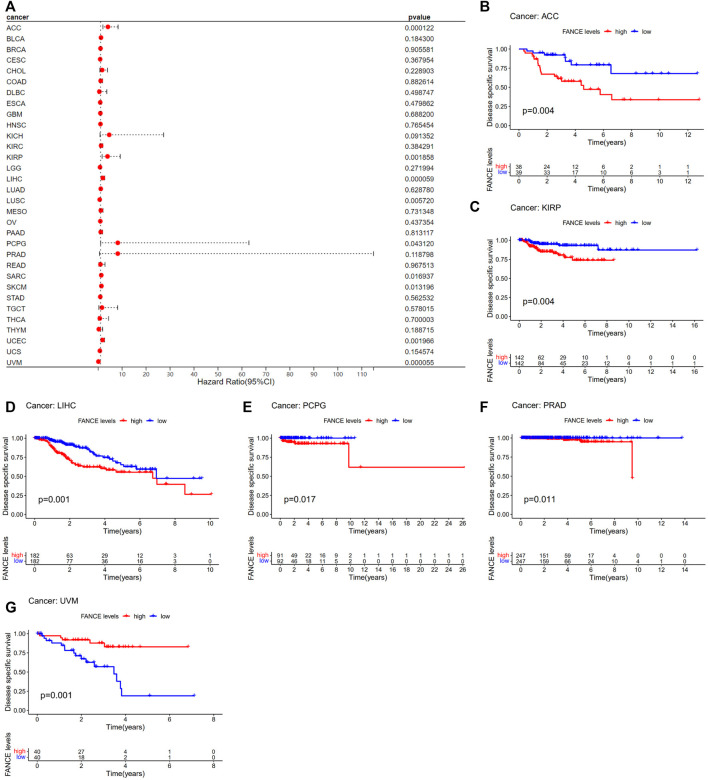
Correlation between *FANCE* expression and disease-specific survival (DSS) using data from the TGCA database. **(A)** Forest plot of *FANCE* expression and DSS in 32 cancers. **(B–G)** The Kaplan–Meier survival analysis for *FANCE* expression and DSS in six cancers. *p < .05; **p < .01; and ***p < .001.

**FIGURE 6:**
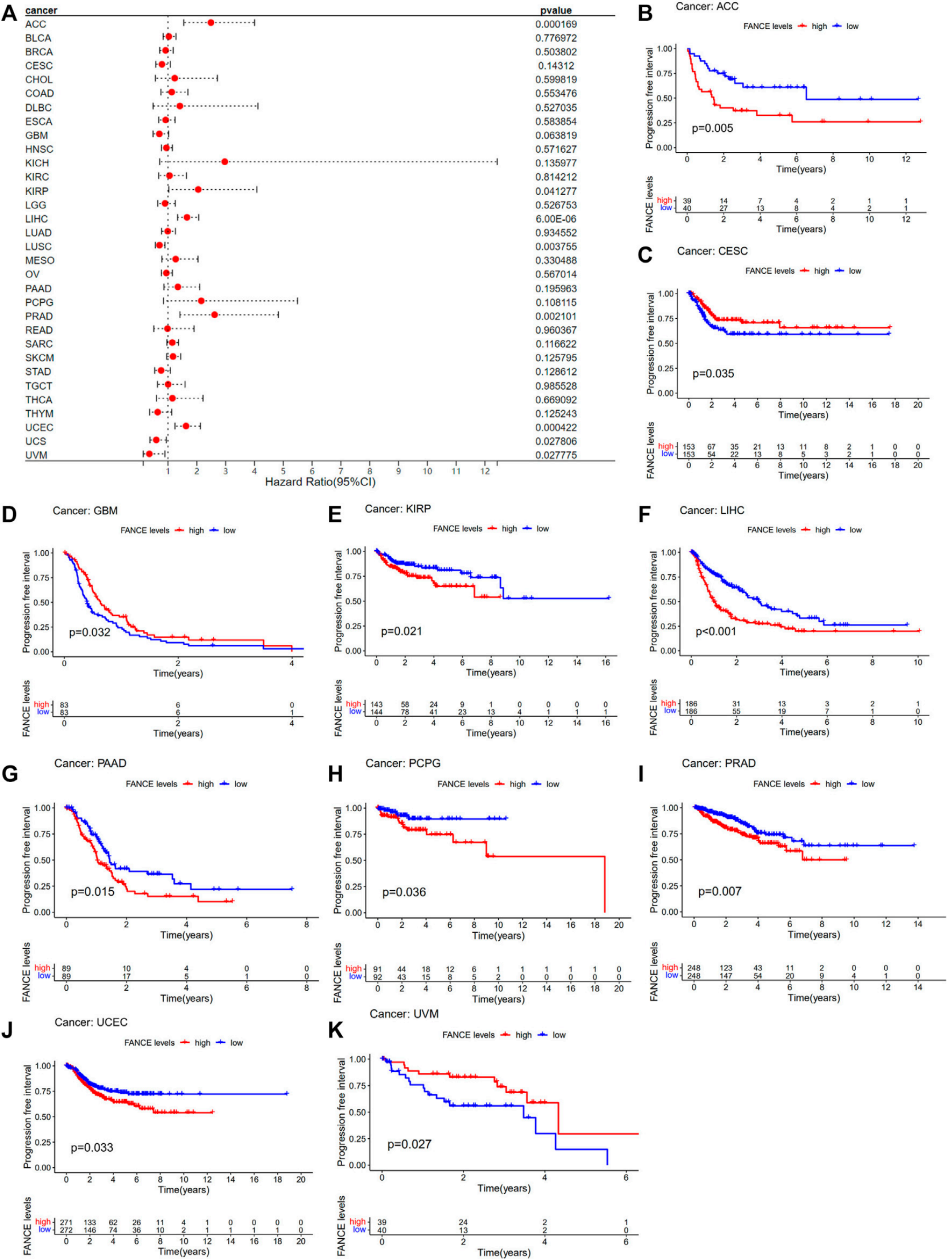
Correlation between *FANCE* expression and progression-free interval (PFI) using data from the TGCA database. **(A)** Forest plot of *FANCE* expression and PFI in 32 cancers. **(B)** The Kaplan–Meier survival analysis for *FANCE* expression and PFI in 10 cancers. *p < .05; **p < .01; and ***p < .001.

**FIGURE 7:**
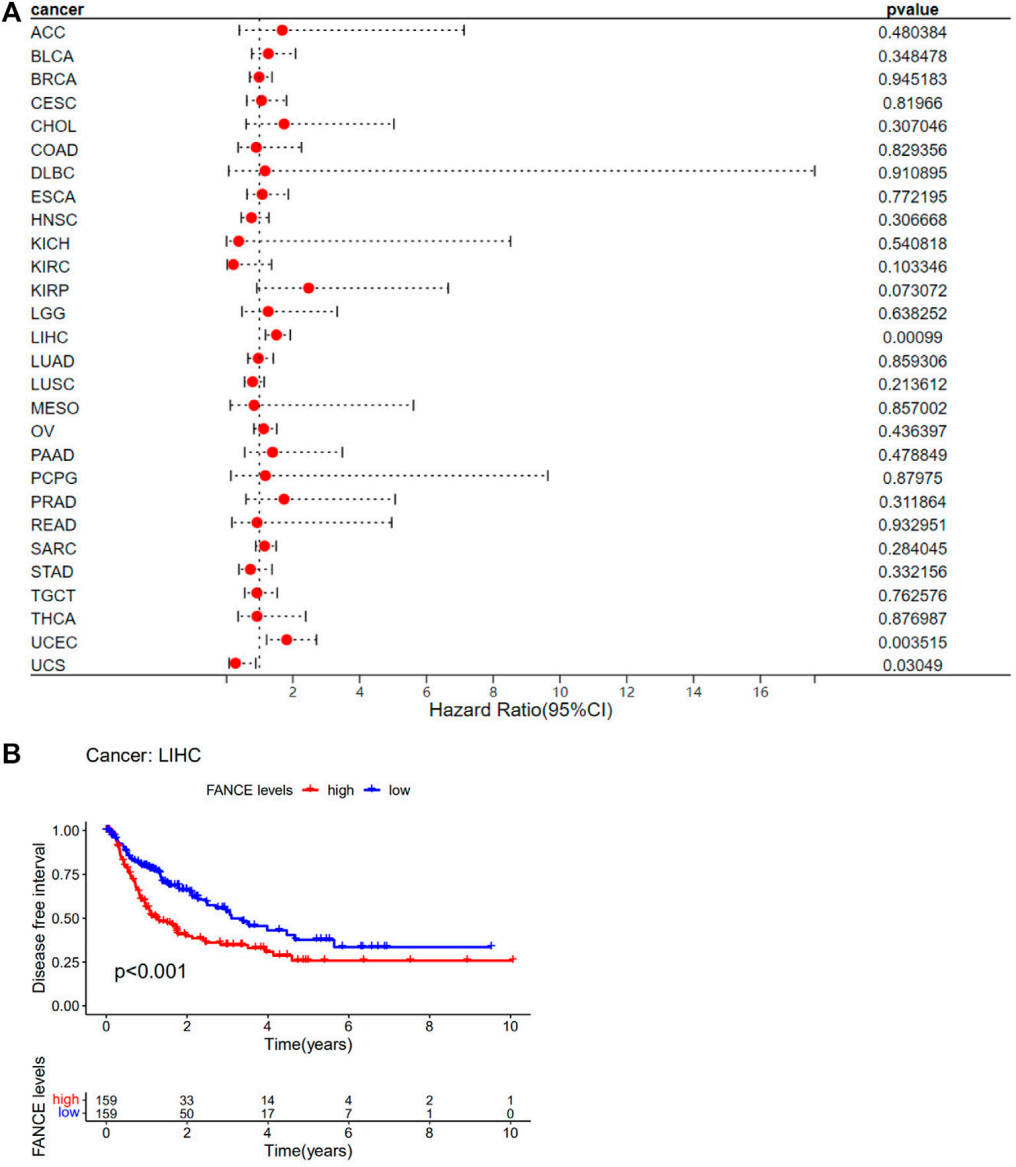
Correlation between *FANCE* expression and disease-free interval (DFI) using data from the TGCA database. **(A)** Forest plot of *FANCE* expression and DFI in 32 cancers. **(B)** The Kaplan–Meier survival analysis for *FANCE* expression and DFI in LIHC. *p < .05; **p < .01; and ***p < .001.

Survival analyses of *FANCE* expression were evaluated using Cox regression analysis and KM curves based on OS, DSS, DFI, and PFI as prognostic indicators. Cox regression analysis showed *FANCE* correlated with OS in UCEC, LIHC, ACC, KIRP, SARC, SKCM as a risky gene (HR > 1), and in LUSC, READ, UVM as a protective gene (HR < 1) (Figure 4A). KM curves indicated patients with high *FANCE* expression had shorter OS in UCEC, LIHC, ACC, CHOL, KIRP, PCPG, SKCM (Figures 4B, D–F, H–J). Conversely, patients with high FANCE expression had longer OS in CESC, LUSC, UVM (Figures 4C, G, K). *FANCE* was associated with DSS in UCEC, LIHC, ACC, KIRP, PCPG, SARC, SKCM (HR > 1), and UVM, LUSC (HR < 1) (Figure 5A). Patients with high *FANCE* expression exhibited shorter DSS in LIHC, PRAD, ACC, KIRP, PCPG (Figures 5B–F), and exhibited longer DSS in UVM (Figures 5G). *FANCE* correlated with PFI in UCEC, LIHC, PRAD, ACC, KIRP as a risky gene (HR > 1), and in UCS, UVM, LUSC as a protective gene (HR < 1) (Figure 6A). PFI was shorter in patients with high *FANCE* expression in UCEC, LIHC, PRAD, ACC, KIRP, PAAD, and PCPG (Figures 6B, E–J), but longer in CESC, GBM, and UVM (Figures 6C, D, K). (Braun et al., 2016) *FANCE* correlated with DFI in UCEC, LIHC (HR > 1), and in UCS (HR < 1) (Figure 7A). LIHC patients with high *FANCE* expression had shorter DFIs (Figure 7B). These results suggest that high expression of *FANCE* affects the prognosis of multiple tumors. It is particularly associated with the shorter survival of UCEC and LIHC and the longer survival of CESC.

**FIGURE 8:**
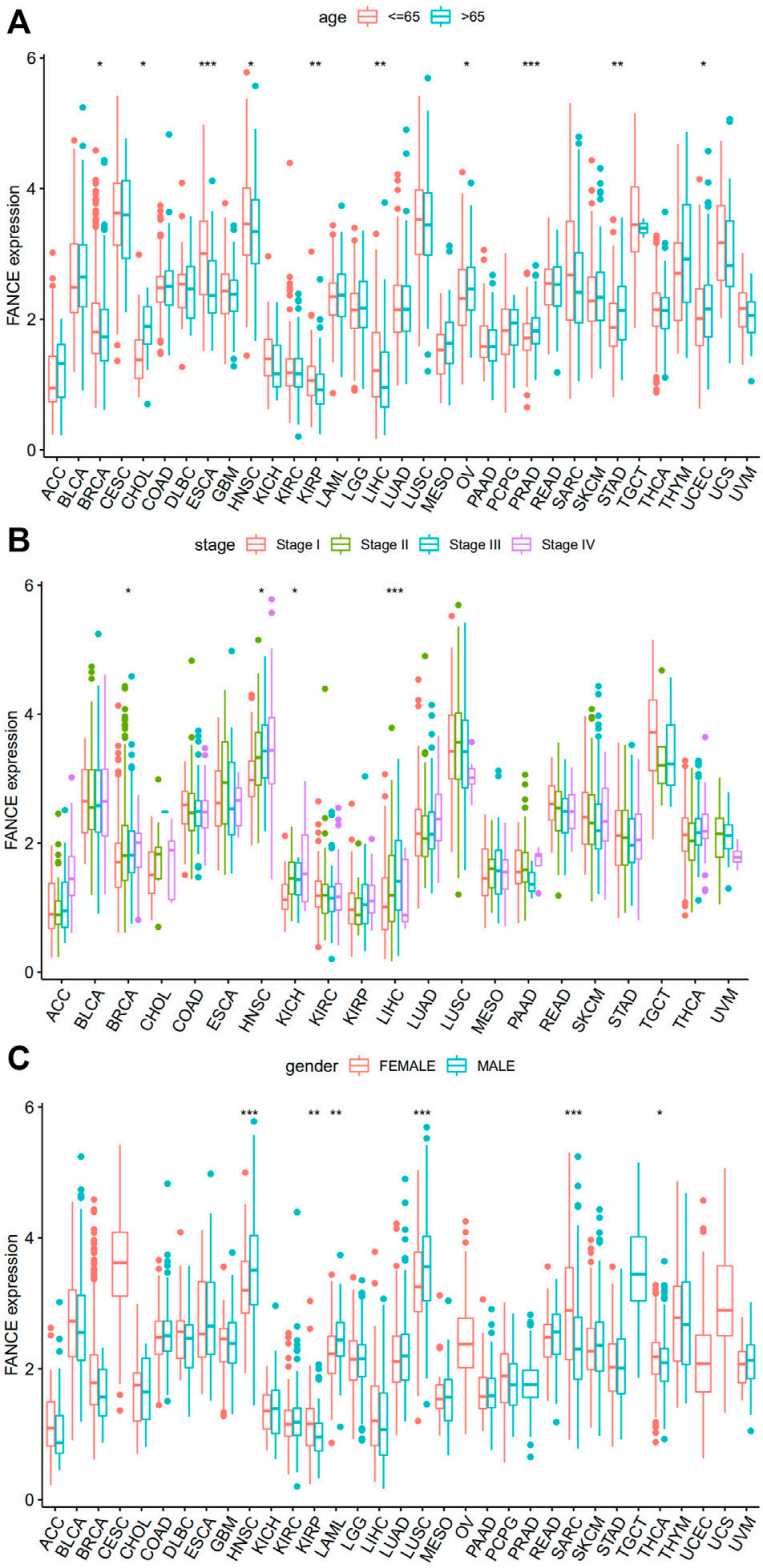
Correlation between *FANCE* expression and **(A)** age, **(B)** stage, and **(C)** gender in 33 cancers from TCGA database. *p < .05; **p < .01; and ***p < .001.

We analyzed *FANCE* and clinical phenotype. Studies have shown people over 65 years old have significantly increased cancer incidence and mortality, such as endometrial cancer and ovarian cancer (Ries, 1993; Yancik, 1993; Braun et al., 2016). We found that patients aged 65 and older had higher *FANCE* expression in OV, UCEC, CHOL, PRAD, and STAD, while expression was lower in BRCA, ESCA, HNSC, KIRP, and LIHC (Figure 8A). We also analyzed whether *FANCE* expression was substantially linked with the TNM stage in LIHC, BRCA, HNSC, and KICH (Figure 8B). Males had higher *FANCE* expression in KIRP, KIRP, and THCA, and females had higher *FANCE* expression in HNSC, LAML, and LUSC (Figure 8C). Further, these findings indicate that *FANCE* expression is clinically significant in a wide variety of tumors.

### 3.4 *FANCE* expression and tumor immunity infiltration

**FIGURE 9:**
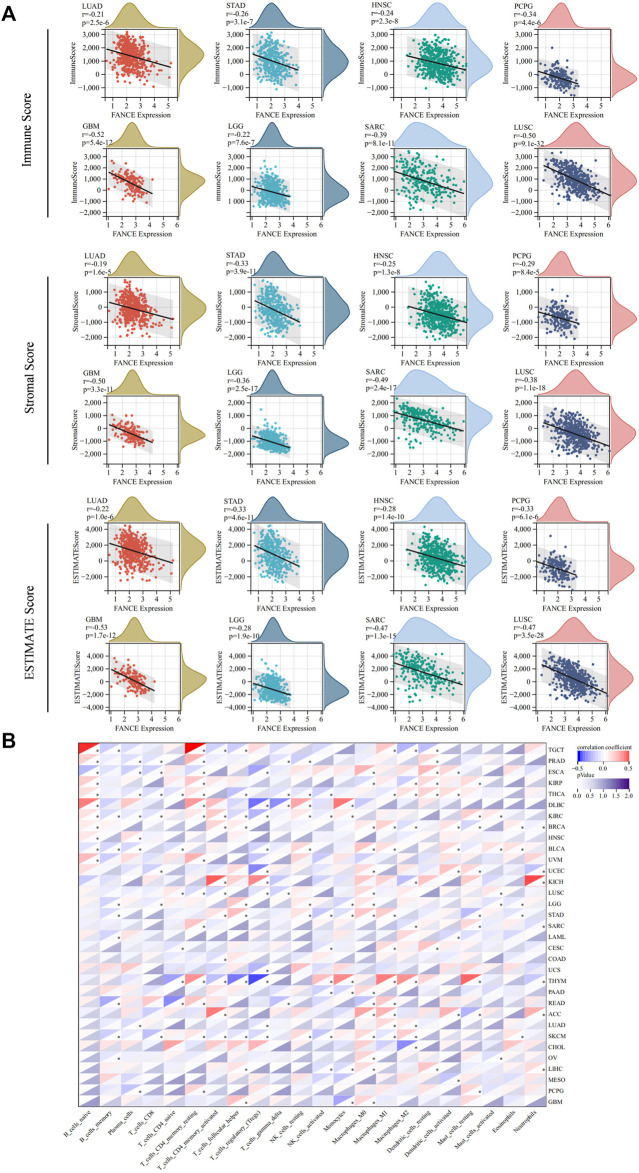
Tumor microenvironment (TME) and tumor-infiltrating immune cells (TIICs) analysis with *FANCE* expression in pan-cancer. **(A)** Correlation between *FANCE* expression and the immune Score, stromal Score, and ESTIMATE score in TME. The top eight cancers with the most significant association were displayed, including LUAD, STAD, HNSC, PCPG, GBM, LGG, SARC, and LUSC. **(B)** Correlation between *FANCE* expression and the proportion of 22 types of TIICs. *p < .05; **p < .01; and ***p < .001.

We analyzed *FANCE* expression and TME, TIICs, immune-regulated genes, and immune checkpoint genes to investigate tumor immunity. We calculated stromal, immune, and estimate scores to analyze *FANCE* expression and TME. Results reveal that *FANCE* expression was significantly negatively correlated with both immune scores and stromal scores in UCEC, OV, LUSC, GBM, SARC, SKCM, HNSC, STAD, LGG, LUAD, PCPG, UVM, and THCA, but was opposite in PRAD. In CESC, LIHC, BRCA, and TGCT, *FANCE* expression was negatively related to stromal scores. The top eight cancers with significant associations between *FANCE* expression and TME were displayed in Figure 9A, including LUAD, STAD, HNSC, PCPG, GBM, LGG, SARC, and LUSC. These indicated that high *FANCE* expression correlates with less stromal and immune cell infiltration, leading to high tumor purity in the vast majority of tumors.

We next analyzed the correlation between *FANCE* expression and the infiltration levels of 22 immune cells. Our results suggest that immune cell infiltration levels were associated with *FANCE* expression in most cancers (Figure 9B). We found diverse correlations between *FANCE* expression levels and different subsets of infiltrating T cells. *FANCE* expression was negatively linked with the infiltration of CD8 T cells in UCEC, LGG, and ESCA. *FANCE* expression was also negatively correlated with the infiltration of Treg cells in UCEC, USC, ESCA, THYM, and DLBC. However, it was positively associated with levels of infiltrating CD4 T cells in ACC, KICH, KIRC, and UVM. Further, *FANCE* expression levels were connected with multiple different subgroups of infiltrating macrophages. *FANCE* expression was positively correlated with the levels of infiltrating M0 macrophages in UCEC, OV, LIHC, LGG, and ACC. It was also positively associated with the levels of infiltrating M1 macrophages in CESC, BRCA, THYM, ACC, and READ. These results suggest that *FANCE* is highly associated with immune cell infiltration in the majority of tumors.

**FIGURE 10:**
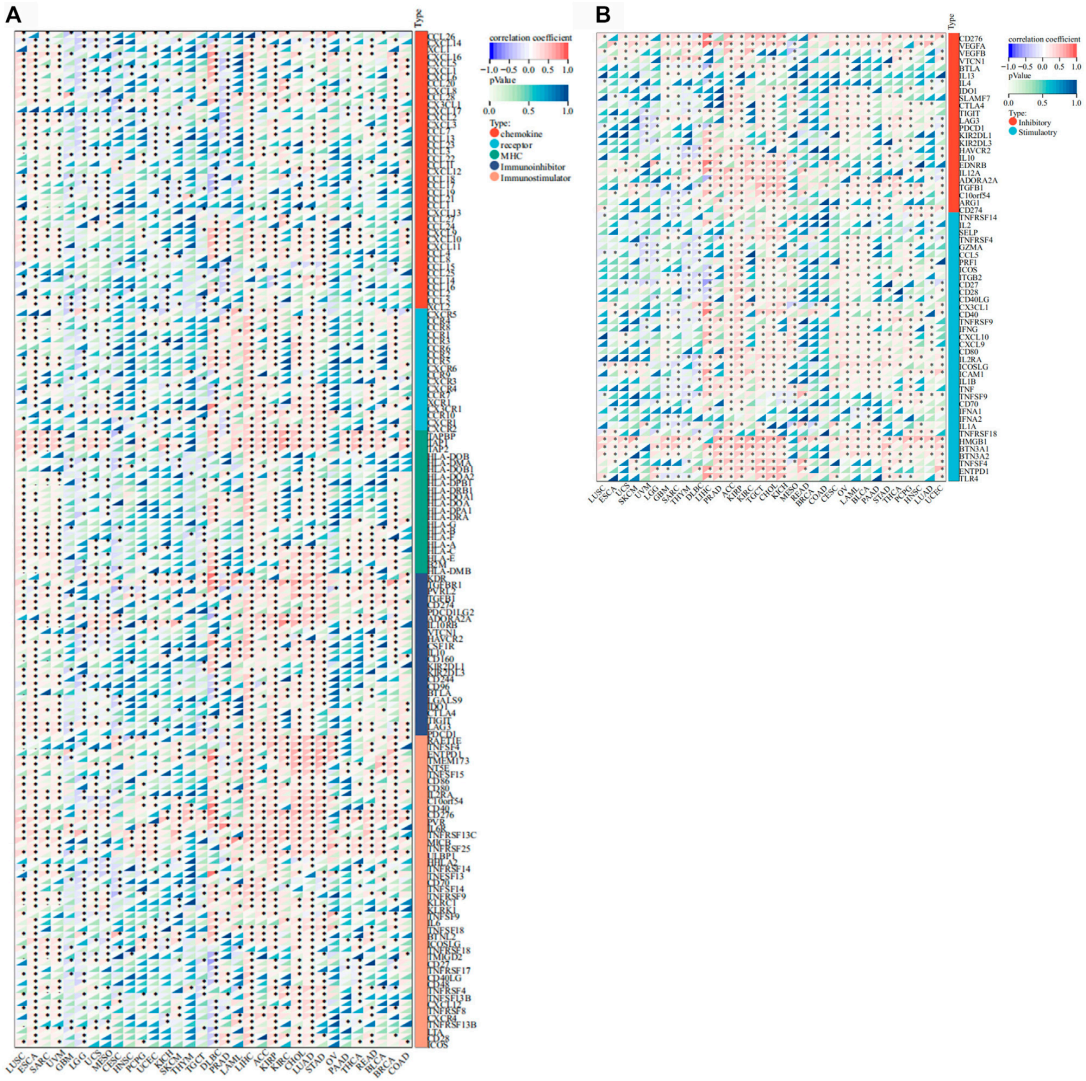
Co-expression of *FANCE* and immune regulated genes. **(A)** Co-expression of *FANCE* and 150 immune moderator genes of five immune pathways (chemokine (41), receptor (18), MHC (21), immunoinhibitor (24), immunostimulator (46)) in 33 cancers. **(B)** Co-expression of *FANCE* with 60 immune checkpoint pathway genes (Inhibitory (24) and Stimulatory (36)) in 33 cancers. *p < .05; **p < .01; and ***p < .001.

We conducted gene co-expression analyses to explore the relationships between *FANCE* expression and immune-related genes in 33 tumors. These genes encoded chemokine, chemokine receptor, MHC, immunoinhibitor, and immunostimulator genes. These results indicated that almost all immune-related genes were co-expressed with *FANCE* (Figure 10A). The majority of immune-related genes were highly associated with *FANCE* in LIHC, ACC, LUAD, STAD, LAML, KIRP, KIRC, and CHOL, while negatively associated with *FANCE* in CESC, GBM, LGG, and TGCT. Results showed MHC genes, including TAPBP, TAP1, and TAP2, were highly positively correlated with *FANCE* in most cancers. Regarding immunoinhibitor genes, *FANCE* expression was significantly positively correlated with KDR, TGFBR1, PVRL2, TGFB1, ADORA2A, and IL10RB. As for immunostimulator genes, *FANCE* was significantly positively linked with C10orf54, CD40, CD276, PVR, IL6R, TNFRSF13C, MICB, TNFRSF25, and ULBP1. These findings suggested that *FANCE* could be involved in regulating tumor immune responses by altering the activity of immune genes.

Furthermore, *FANCE* correlated positively with the majority of immune checkpoint genes in CESC, OV, UCEC, LIHC, BRCA, KIRP, KIRC, ACC, TGCT, BLCA, STAD, PCPG, HNSC, LUAD, while negatively correlating with UCS, GBM, LGG, UVM, THYM, DLBC. *FANCE* was significantly positively linked with the immune checkpoint stimulatory genes HMGB1, BTN3A1, and BTN3A2. As for immune checkpoint inhibitory genes, *FANCE* was significantly positively correlated with CD276, VEGFA, and TGFB1 (Figure 10B). The results indicated that *FANCE* contributes to regulating the immune checkpoint inhibitor (ICI) therapy response in these cancers. *FANCE* was linked to ICIs containing PD-L1/CD274, PD-1/PDCD1, and CTLA4 in UCEC, OV, BRCA, HNSC, STAD, LUAD, TGCT, LTHC, KIRP, KIRC. In CESC, *FANCE* was positively associated with PD-L1 and CTLA4. The results indicated that *FANCE* regulation of ICI therapy response might be related to cancer prognosis in these cancers.

### 3.5 *FANCE* expression and tumor immunotherapy responses

**FIGURE 11:**
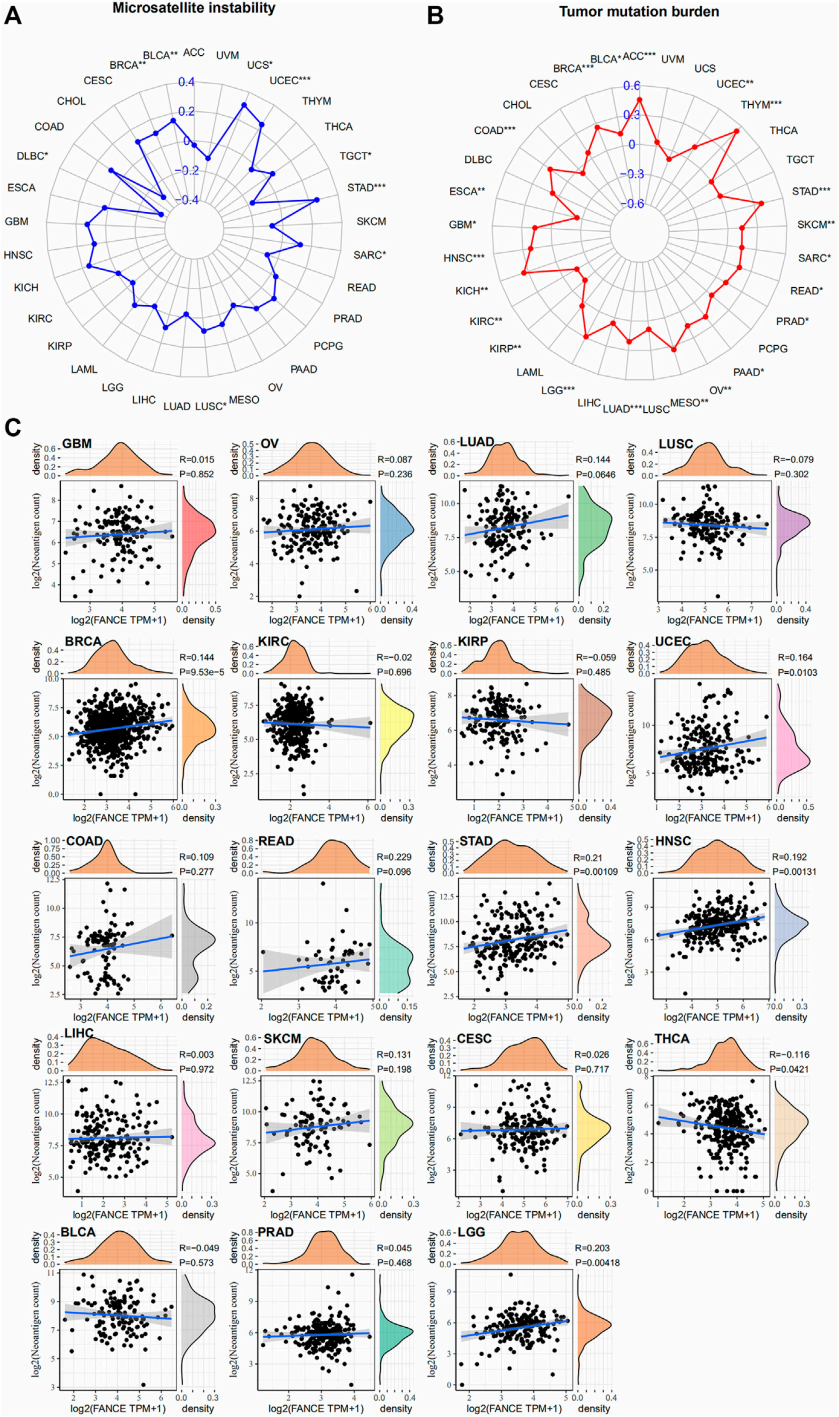
Correlation between *FANCE* expression and microsatellite instability (MSI) **(A)**, tumor mutation burden (TMB) **(B)**, and neoantigens **(C)** analysis in pan-cancer. *p < .05; **p < .01; and ***p < .001.

We further analyzed *FANCE* expression and MSI, TMB, and neoantigens to predict the tumor immunotherapy responses. There are important connections between MSI and ICI therapy sensitivity. Higher TMB results in more neoantigens, increased T cell recognition, and better ICI therapy outcomes (Jardim et al., 2021). Neoantigens were used as markers in cancer immunotherapy that enhance T cell reactivity against these antigens (Schumacher and Schreiber, 2015). *FANCE* expression was positively associated with MSI in seven cancers including UCEC, UCS, STAD, BRCA, BLCA, LUSC, SARC, and negatively in DLBC, TGCT (Figure 11A). *FANCE* expression was positively correlated with TMB in 22 cancers, including UCEC, OV, ESCA, STAD, PRAD, BRCA, PAAD, ACC, BLCA, COAD, GBM, HNSC, KICH, KIRC, KIRP, LGG, LUAD, MESO, READ, SARC, SKCM, THYM (Figure 11B). *FANCE* correlated positively with neoantigens in UCEC, STAD, BRCA, HNSC, LGG, and negatively in THCA (Figure 11C). Results showed *FANCE* expression was highly positively associated with MSI, TMB, and neoantigens in UCEC, BRCA, and STAD especially. The results indicate *FANCE* may regulate ICI therapy response and inspire new therapies development for these cancers.

### 3.6 ScRNA-seq data analysis identified the expression and function of *FANCE*

**FIGURE 12:**
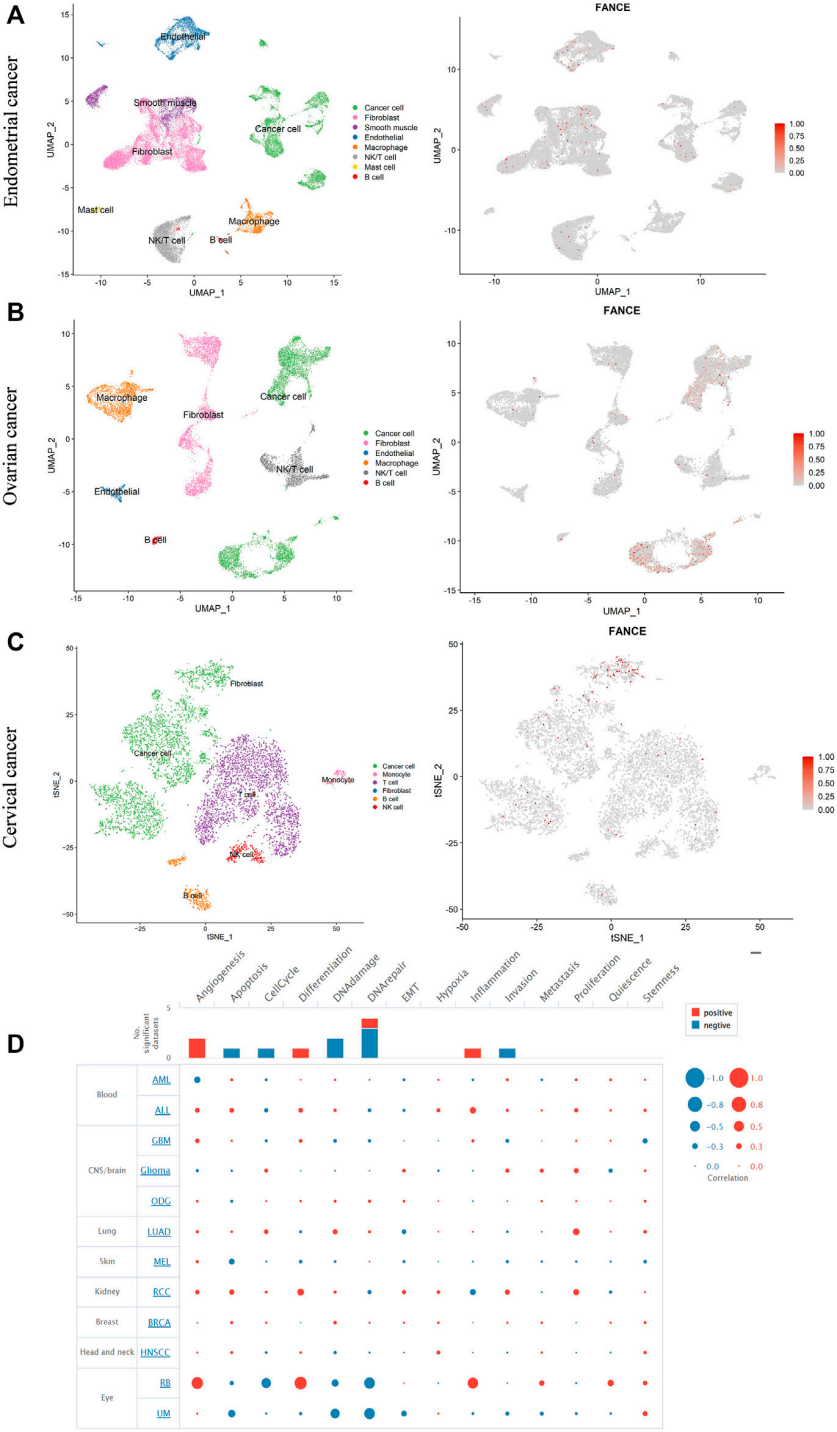
Single-cell RNA sequencing data analyze the expression of *FANCE* in different cells in endometrial cancer **(A)**, ovarian cancer **(B)**, and cervical cancer **(C)** based on the Gene Expression Omnibus (GEO) database. **(D)** The function of FANCE at the single cell level investigated by the cancer single-cell atlas (CancerSEA) database.

We further evaluated *FANCE* expression in different cells of the tumor microenvironment using single-cell RNA sequencing data from the GEO database. Three cancers, including endometrial cancer, cervical cancer, and ovarian cancer, with correlations with clinical phenotype and tumor immunity, were further analyzed. Our results showed that in endometrial cancer, *FANCE* was expressed in cancer cells, fibroblasts, endothelial cells, and T cells (Figure 12A). In ovarian cancer, *FANCE* was predominantly present in cancer cells, with a minority distributed in fibroblasts, T cells, and macrophages (Figure 12B). In cervical cancer, *FANCE* was mainly expressed in cancer cells and less abundant in T cells (Figure 12C). These are consistent *FANCE* expressions that correlate negatively with TME in UCEC, CESC, and OV. *FANCE’s* role in tumor immunity appears to be achieved by altering the function of cancer cells in these cancers. At the immune cell level, it may be mediated by T cells and macrophages. In addition, we explored that *FANCE* functions in cancers by downregulating DNA repair and DNA damage, and upregulating angiogenesis at the single-cell level using the CancerSEA database (Figure 12D).

### 3.7 *Fanc*e gene mutant mice increased tumor susceptibility

**FIGURE 13:**
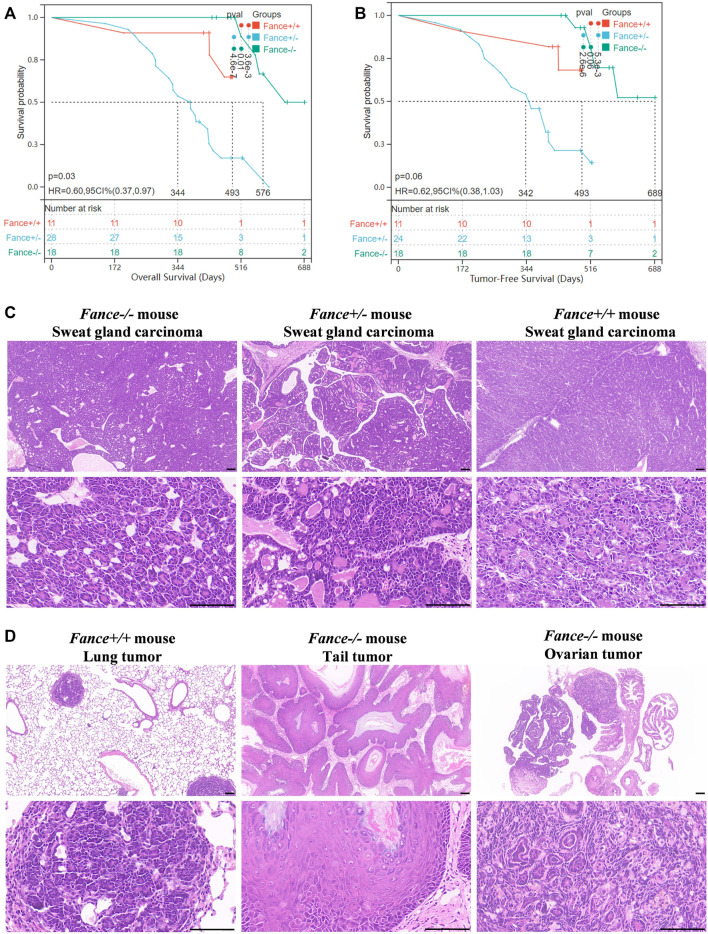
Survival curves and histology of carcinomas in *Fance* homozygous mice, *Fance* heterozygote mice, and wild-type mice with FVB/N background. Overall survival curves **(A)** and tumor-free survival curves **(B)** of the *Fance* ^*−/−*^, *Fance* ^*+/-*^, and *Fance* ^*+/+*^ mice cohort. *p* values were determined by the Log-rank test. *p* < .05 was considered significantly different. **(C)** H&E-stained paraffin-embedded sections of sweat gland carcinoma in *Fance* ^*−/−*^, *Fance* ^*+/-*^, and *Fance* ^*+/+*^ mice cohort. **(D)** Histology of tumors located at lung in *Fance* ^*+/+*^ mice. Histology of tumors located at tail and ovary in *Fance* ^*−/−*^ mice. All scale label = 100 um.

FA genes were associated with cancer predisposition. Especially mutations in *BRCA1/FANCS* and *BRCA2/FANCD1* caused ovarian cancer and breast cancer in women. We generated a cohort of female *Fance* homozygous mutant (*Fance* ^*−/−*^) mice, *Fance* heterozygous mutant (*Fance* ^*+/−*^) mice, and wild-type (*Fance* ^*+/+*^) mice for long-term monitoring to determine tumor prone of *Fance* mutant. Tumors (93.5% were sweat gland carcinomas) were detected in 85.7% (24/28) of *Fance* ^*+/−*^ mice, 27.3% (3/11) of *Fance* ^*+/+*^ mice, and 22.2% (4/18) of *Fance* ^*−/−*^ mice (Table 1; Figure 13C). *Fance* ^*+/−*^ mice had increased tumor incidence than *Fance* ^*−/−*^ and *Fance* ^*+/+*^ mice (*p* < .05). Survival analysis showed that *Fance* ^*+/−*^ had shorter OS and tumor-free survival (TFS) than *Fance* ^*−/−*^ and *Fance* ^*+/+*^ mice (*p* < .05) (Figures 13A, B). The OS of *Fance* ^*+/+*^ mice was shorter than *Fance* ^*−/−*^ mice (*p* < .05). Two *Fance* ^*−/−*^ mice had tumors located at their tails, and one had ovarian tumors. A *Fance* ^*+/+*^ mouse detected lung tumors with sweat gland carcinoma (Figure 13D). The liver, spleen, stomach, kidney, ovary, and skin tissues of other mice found no obvious abnormalities (Supplementary Figure S2). These results indicated that *Fance* ^*+/−*^ mice had increased carcinogenesis and shorter survival. Deeper studies are needed to prove results definitively.

**TABLE 1:** Increased cancer incidence in *Fance* ^*−/−*^, *Fance* ^*+/−*^, and *Fance* ^*+/+*^ female mice

Genotype	Background	Tumor type
*Fance* ^*−/−*^ (*n* = 18)	FVB/N	2 sweat gland carcinoma
		2 tail tumor
		1 ovarian tumor
*Fance* ^*+/*−^ (*n* = 28)	FVB/N	24 sweat gland carcinoma
*Fance* ^*+/+*^ (*n* = 11)	FVB/N	3 sweat gland carcinoma
		1 lung tumor

## 4 Discussion

FA genes play an influential role in tumor prognosis and immunity. However, the role of *FANCE* in tumors remains uncertain. We performed a pan-cancer analysis of *FANCE* expression with the prognostic and immunological role in 33 different cancers based on information extracted from databases. Additionally, we explored the tumorigenesis phenomenon of *Fance* using mutant mice.

*FANCE* may serve as a prognostic biomarker. Our results suggest that *FANCE* has clinical associations with a variety of cancers. High *FANCE* expression correlated with poor prognosis in UCEC, LIHC, ACC, CHOL, KIRP, PCPG, SKCM, PRAD, and PAAD, while it correlated with better prognosis in CESC, LUSC, and UVM. We found *FANCE* expression correlated with shorter OS and PFI in UCEC and longer OS and PFI in cervical cancer. A previous study showed *FANCE* was an independent factor in predicting poor prognosis in LIHC (Takahashi et al., 2022). Our data suggested LIHC patients with high *FANCE* (regarded as a risky gene) expression had shorter OS, DSS, DFI, and PFI. These results were consistent with previous results. Patients with the *FANCE* variant had better prognosis for non-small cell lung cancer (Mo et al., 2022). We found high *FANCE* expression in LUSC was associated with longer OS and served as a protective factor in OS, DSS, and DFI. Moreover, *FANCE* served as a risky gene and correlated with shorter survival in UCEC (shorter OS and PFI) and PRAD (shorter PFI). We discovered patients over 65 had higher *FANCE* expression in UCEC and PRAD. These results indicated that elevated *FANCE* expression may be related to increased mortality in these cancers. While we found high *FANCE* expression correlated with longer OS and PFI in CESC and UVM. The expression and gene activity scores of *FANCE* were higher in CESC than in normal tissues. The role of *FANCE* in cervical cancer should be further studied. Further, patients with FA gene defects were more likely to develop human papillomaviruses (HPV) associated squamous cell carcinoma at multiple anatomical sites, including the HNSC (Kutler et al., 2003a; Kutler et al., 2003b; Lowy and Gillison, 2003; Rosenberg et al., 2003). HPV was identified as a significant positive prognostic factor for females with some solid tumors (Preissner et al., 2022). Our study found that females have higher *FANCE* expression in HNSC, these may help explain the lower incidence compared to males (Carazo-Casas et al., 2022).

*FANCE* expression regulated TIICs infiltration related to cancer prognosis. CD8 T cells exert cytotoxic effects on tumor cells by activating their apoptosis mechanisms (Farhood et al., 2019). FA genes significantly prolong the OS of cervical cancer by promoting the infiltration of CD8 T cells and memory T cells (Wang et al., 2021). Treg cells suppress aberrant immune responses and anti-tumor immune responses and are associated with poor prognosis (Tanaka and Sakaguchi, 2017). Our analysis of endometrial cancer scRNA-seq results showed that *FANCE* was expressed in T cells. High *FANCE* expression in endometrial cancer was correlated with reduced CD8 T cells and raised Treg cells infiltration. These may be related to poor prognosis. Previous studies have shown that fibroblasts maintain frequent communication with cancer cells to promote the development of endothelial cancer (Yu et al., 2022). We also found *FANCE* is predominantly expressed in fibroblasts and correlated with poor prognosis in endometrial cancer according to scRNA-seq analysis. M1 macrophages enhance cytotoxicity and activate cytotoxic T cells to eliminate tumor cells, while M2 macrophages contribute to cancer development (Chen et al., 2018). High *FANCE* expression in cervical cancer was positively associated with M1 macrophage infiltration, consistent with its favorable prognosis. According to our scRNA-seq analysis of cervical cancer cells, *FANCE* was expressed mainly in cancer cells and less in T cells. As a result, more research is needed to explore the relationship between *FANCE’s* effect on the immune microenvironment and its prognostic value in cervical cancer.

*FANCE* expression was associated with immunotherapy responses. PD-1 and CTLA-4 immune checkpoint blockers have been shown in studies to improve the survival of patients with recurrent or metastatic cervical cancer (Allouch et al., 2020; Ferrall et al., 2021). In cervical cancer, *FANCE* expression was positively correlated with PDL1 and CTLA4. Binding to PD-L1 ligand, PD-1 inhibits intracellular signaling in effector T cells and Treg cells, mediates T cell apoptosis, or induces T cell dysfunctional states, which decrease tumor immunity and promote tumor survival (Keir et al., 2008; Wei et al., 2017). T cells release CTLA4, which binds to antigen-presenting cells B7-1/B7-2 (Tai et al., 2012). Blockade of CTLA4 promotes therapeutic response for T cell activation against tumor cells (Waldman et al., 2020). It suggested ICI therapy may be effective and promote a better prognosis for cervical cancer with high expression of *FANCE*. Blockade of PD-1 and PD-L1 immune checkpoints led to better outcomes in high-grade serous ovarian cancer and recurrent or metastatic endometrial cancer (Post et al., 2020; Wan et al., 2021). *FANCE* expression in ovarian cancer and endometrial cancer might enhance ICIs therapy response since positively correlated with PD-1 and PD-L1.

*FANCE* expression might also enhance immunotherapy response due to its positive relation to MSI, TMB, and neoantigens. *FANCE* expression was positively correlated with MSI in seven cancers, especially in endometrial cancer and gastric cancer. Nearly 30% of primary endometrial cancer were MSI-high (MSI-H). PD-1 inhibitor is more effective against MSI-H than against microsatellite stable (MSS) in advanced endometrial cancers (Green et al., 2020). Approximately 4%–20% of gastroesophageal cancer exhibit MSI, and ICIs therapy was effective against MSI-H tumors (van Velzen et al., 2020; Joshi and Badgwell, 2021). We hypothesize *FANCE* expression enhances the effectiveness of immunotherapy in cancers with positive MSI. The objective response rate of anti-PD-1 or anti-PD-L1 therapy in 55% of cancers was positively correlated to TMB, in a study involving 27 cancers (Yarchoan et al., 2017). In a study of 1,48,803 tumor samples, MSS/TMB-high tumors were more prevalent than MSI-H cancers and benefited more from immunotherapy (Goodman et al., 2019). As a result of our research, *FANCE* expression was positively correlated with TMB in 22 cancers containing endometrial cancer, gastric cancer, ovarian cancer and breast cancer, and so on. Immunotherapies might be more effective in these cancers. *FANCE* expression positively correlated with neoantigens in endometrial cancer, gastric cancer, ovarian cancer, and breast cancer. These results may inspire the development of immunotherapies that selectively activate T cells against these antigens.

FA gene mutations contributed to tumors including squamous cell carcinoma, breast cancer, and ovarian cancer (Milletti et al., 2020). Tumorigenesis is also observed in FA mutant animal models. Primary cancers have been observed in *Fanca*, *Fancm*, *Fancf*, *Fancd1*, and *Fancd2* mutant mice, including ovarian adenocarcinomas, hepatocellular carcinomas, lymphomas, ovarian granulosa cell tumors, spindle-cell sarcomas, lung carcinomas, breast cancers, and histiocytic sarcomas (Houghtaling et al., 2003; Wong et al., 2003; Bakker et al., 2009; Bakker et al., 2012; Luo et al., 2014; Hartford et al., 2016). We observed tumors including sweat gland carcinomas, ovarian tumors, lung tumors, and tail tumors in *Fance* ^*−/−*^, *Fance* ^*+/−*^, and *Fance* ^*+/+*^ mice. *Fance* ^*+/−*^ mice had increased tumor incidence and shorter survival than *Fance* ^*−/−*^ and *Fance* ^*+/+*^ mice. The mechanisms of carcinogenesis associated with a dysregulation FA pathway are complicated, as FA proteins contribute to genome homeostasis associated with DNA repair and cell division proteins (Nalepa and Clapp, 2018). We speculate heterozygous mutations in the *Fance* gene cause its dysfunction to maintain genome homeostasis, resulting in increased tumorigenic responses and promoting tumor progression. Further studies are needed to understand the molecular mechanisms involved.

Our study had several limitations. First, we examined *FANCE* expression with prognosis, tumor immunity infiltration, and immunotherapy using bioinformatics methods. However, we lack *in vitro* experimental validation. Secondly, we examined tumorigenesis performed in a cohort of *Fance* mutant mice, while the sample size was relatively small and further verification requires for diagnosis and prognostic correlation analysis. Finally, the molecular mechanism of *Fance* tumorigenesis is complex and requires further investigation. The results of our bioinformatics analysis and validation by *Fance* mutant mice were only recommendations for future development.

In conclusion, this study systematically described the role of *FANCE* in different cancer types. Our results suggest that *FANCE* is correlated with clinical prognosis, immune infiltration, and immunotherapy in a variety of cancers. Further research is needed to verify the specific mechanisms involved. This study illustrates the role of *FANCE* in tumor development and provides a theoretical basis for more precise treatment in the future. In addition, the *Fance* mutant mouse model also offers potential value for more in-depth mechanistic exploration in the future.

## Data Availability

The datasets presented in this study can be found in online repositories. The names of the repository/repositories and accession number(s) can be found in the article/Supplementary Material.

## Supplementary material

SUPPLEMENTARY FIGURE S1 Gene set enrichment analysis of *FANCE* expression in cancers. The top 5 most relevant signaling pathways in the KEGG dataset were displayed.

SUPPLEMENTARY FIGURE S2 Histology of liver, spleen, stomach, kidney, ovary, and skin tissues of Fance−/−, Fance+/−, and Fance+/+ female mice cohort and found no significant abnormalities. All scale label = 100 um.
