# Supplementary figures and images for: Pan-cancer analysis of the prognostic and immunological role of Fanconi anemia complementation group E
Source: Front Genet. 2023 Jan 4;13:1024989. doi: 10.3389/fgene.2022.1024989 (PMC9846156; doi:10.3389/fgene.2022.1024989)

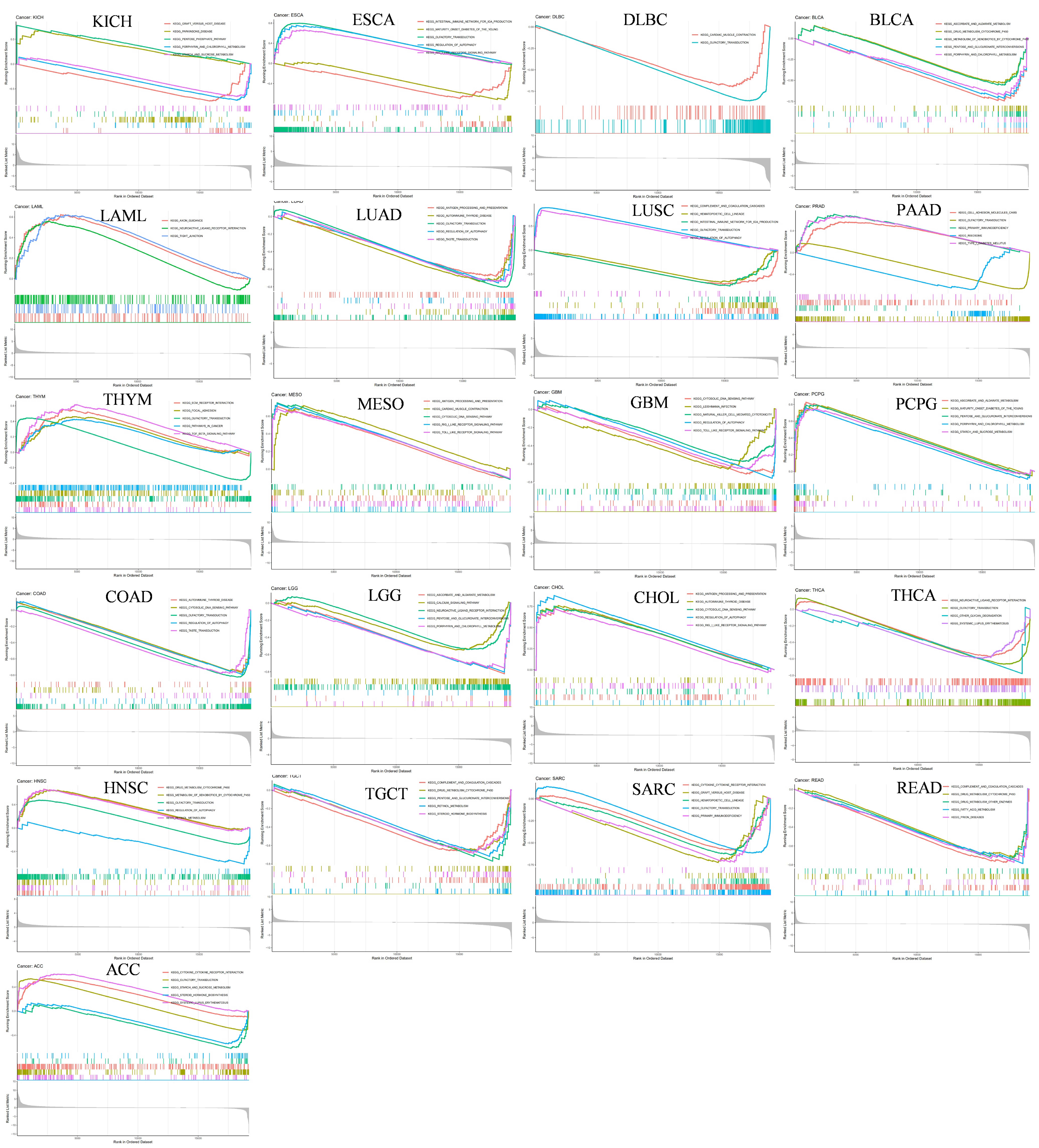

Supplement: Supplementary file 1 [file Image1.JPEG]

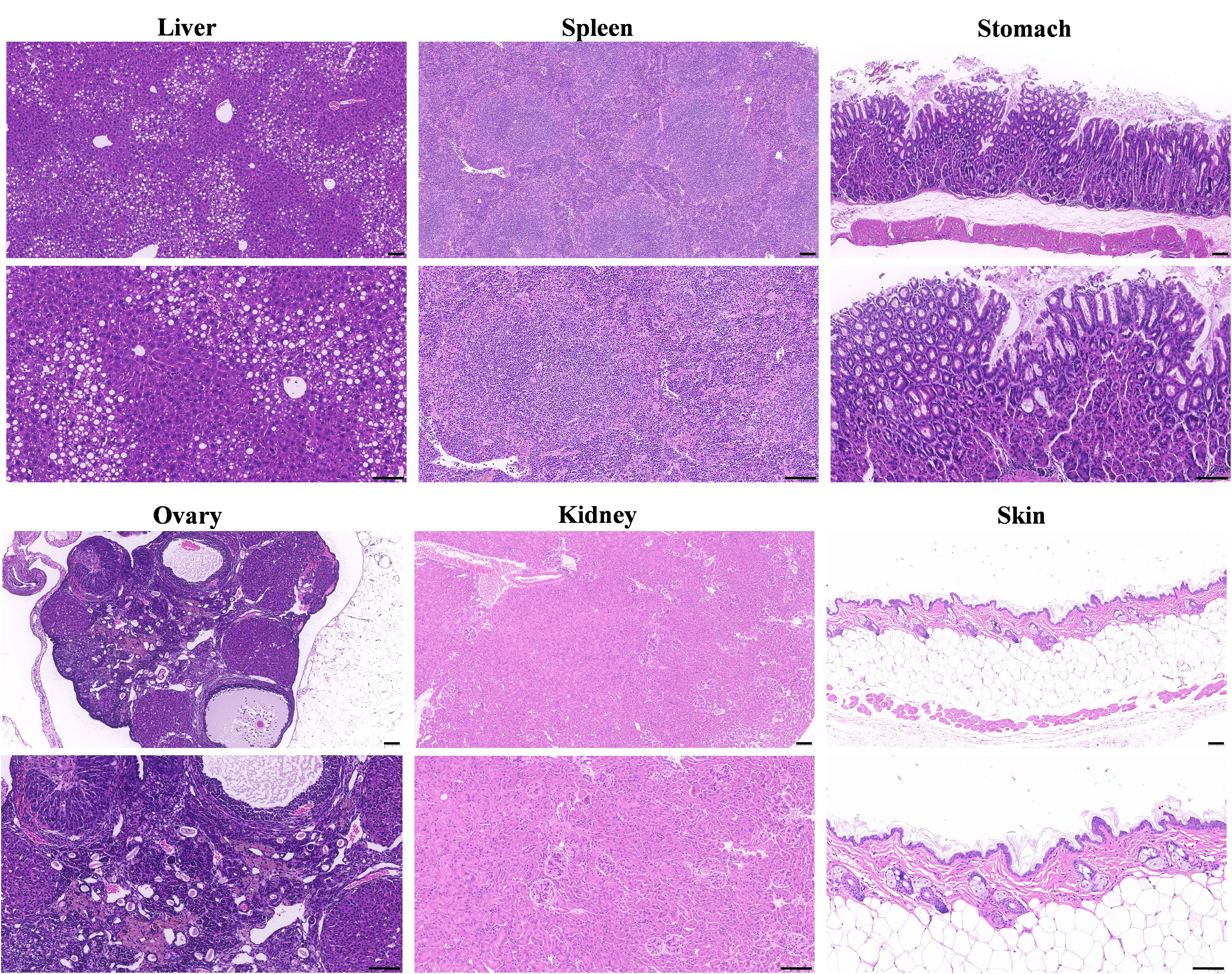

Supplement: Supplementary file 2 [file Image2.JPEG]
